# Taxonomic filtering accompanies functional expansion during long-term soil restoration

**DOI:** 10.1093/ismejo/wrag131

**Published:** 2026-05-22

**Authors:** Tim Goodall, Susheel Bhanu Busi, Briony Jones, Amy Thorpe, Robert I Griffiths, John Redhead, Lucy Hulmes, Sarah Hulmes, Lucy Ridding, Jodey Peyton, Gloria Pereira, Hyun Soon Gweon, Daniel S Read, Richard Pywell

**Affiliations:** UK Centre for Ecology & Hydrology, Wallingford, OX10 8BB, United Kingdom; UK Centre for Ecology & Hydrology, Wallingford, OX10 8BB, United Kingdom; UK Centre for Ecology & Hydrology, Bangor, LL57 2UW, United Kingdom; UK Centre for Ecology & Hydrology, Wallingford, OX10 8BB, United Kingdom; School of Environment, Bangor University, Bangor, LL57 2UR, United Kingdom; UK Centre for Ecology & Hydrology, Wallingford, OX10 8BB, United Kingdom; UK Centre for Ecology & Hydrology, Wallingford, OX10 8BB, United Kingdom; UK Centre for Ecology & Hydrology, Wallingford, OX10 8BB, United Kingdom; UK Centre for Ecology & Hydrology, Wallingford, OX10 8BB, United Kingdom; UK Centre for Ecology & Hydrology, Wallingford, OX10 8BB, United Kingdom; UK Centre for Ecology & Hydrology, Lancaster, LA1 4AP, United Kingdom; School of Biological Sciences, University of Reading, Reading, RG6 6AS, United Kingdom; UK Centre for Ecology & Hydrology, Wallingford, OX10 8BB, United Kingdom; UK Centre for Ecology & Hydrology, Wallingford, OX10 8BB, United Kingdom

**Keywords:** calcareous grassland restoration, land-use chronosequence, microbial succession, soil legacy effects, carbon cycling, above–belowground decoupling

## Abstract

The restoration of species-rich calcareous grasslands is a critical conservation objective, yet the recovery of the invisible below-ground microbiome remains poorly quantified compared to above-ground vegetation. Using a unique 143-year land-use chronosequence on Salisbury Plain, UK, we investigated the trajectory of ecosystem reassembly across arable, regenerating (23 and 67 years), and ancient grasslands. By integrating vegetation surveys with soil physiochemistry, microbial profiling, and shotgun metagenomics, we identified a decoupling between floral and edaphic recovery. While the diversity of vegetation recovered relatively rapidly, approaching ancient grassland levels within 23–67 years, soil properties exhibited persistent legacy effects and slow convergence. Bacterial richness decreased with restoration age; this taxonomic contraction was conversely matched by an expansion in inferred metagenomic functional potential. This was reflected in increased functional gene richness and shifts in the relative abundance of specific SEED-annotated functions towards metabolic pathways associated with complex carbon cycling and stress tolerance. These shifts were congruent with the emergence of specific, unnamed genera belonging to *Pseudomonadota* and *Actinomycetota*, and the *Bacillota* species *Pristimantibacillus*. The soil ecosystem remained distinct from the 143-year stage even after 67 years of recovery, characterized by persistent legacy phosphorus and a slow accumulation of soil organic matter. These findings suggest that passive regeneration alone may be insufficient for full soil functional recovery, and that strategies targeting microbial assembly and long-term carbon dynamics warrant further evaluation.

## Introduction

Calcareous grasslands are among the most species-rich ecosystems in Northern Europe, supporting exceptional floral diversity and providing critical ecosystem services, including carbon sequestration and hydrological regulation [1]. However, the extent and quality of these habitats have declined precipitously over the last century due to agricultural expansion and intensification [2]. This conversion to agricultural land, typically involving deep tillage and the application of inorganic fertilizers, fundamentally alters the soil’s physicochemical architecture and disrupts the complex biotic interactions of the below-ground microbiome [3]. While the immediate impacts of agricultural intensification are well documented [4], the capacity for these ecosystems to recover following the cessation of intensive agricultural practice, using the approach of passive restoration [5] through natural regeneration [6], remains a subject of significant ecological debate [7].

Restoration studies across terrestrial ecosystems have shown that biodiversity recovery is often incomplete relative to reference systems [7], even where restoration increases diversity and reduces degradation effects. This pattern is particularly relevant in post-agricultural landscapes, where historical land use can leave persistent soil legacies that continue to shape community assembly long after visible vegetation recovery has begun [8]. Studies from a range of restored systems have shown that microbial communities may remain distinct from reference states for years to decades, with elevated nutrient pools, especially phosphorus, implicated as one mechanism limiting recovery [5]. These studies suggest that post-agricultural restoration should be understood not simply as vegetation reassembly, but as a longer-term reorganization of soil biotic and abiotic states.

A central uncertainty in restoration ecology is the degree of hysteresis present in soil systems. Above-ground vegetation communities may begin to visibly reassemble once disturbance pressures are removed [9], and soil ecosystems often exhibit a legacy effect [10, 11]. Anthropogenic inputs, particularly elevated levels of phosphorus and nitrogen, can persist for decades [12], potentially locking the ecosystem into an alternative trajectory, inhibiting the return of characteristic grassland biodiversity [13]. It is unclear whether the recovery of soil microbial diversity and function tracks linearly with vegetation recovery or if it operates on a decoupled, much slower trajectory. Understanding these temporal dynamics is critical, as microorganisms mediate key biogeochemical cycles that underpin the long-term stability of the restored ecosystem. The need to distinguish above-ground from below-ground recovery is reinforced by a wider literature showing that plant–soil interactions [14] and soil legacies operate across multiple spatial and temporal scales, from rapid rhizosphere responses to long-term shifts in organic matter quality, nutrient availability, and microbial community structure [10]. These dynamics are especially relevant in restoration studies based on chronosequences, which provide rare access to long timescales but require caution when inferring mechanism from spatial substitution alone [15]. Chronosequence approaches remain highly informative for identifying broad successional patterns in soils and microbial communities, if interpretation remains focused on trajectories and associations rather than direct proof of process.

Our study investigates these dynamics using a unique 143-year chronosequence located on Salisbury Plain in the UK [11]. This landscape serves as a rare ‘space-for-time’ substitution experiment, where historical land-use records allow us to identify areas of calcareous grassland with distinct cessation dates of agricultural practices. By analysing soils from active arable land (0 years) and comparing them to grasslands regenerating for 23 years (recent), 67 years (middle), and >143 years (old/ancient), we can quantify the rate and direction of edaphic functional change and functional expansion. Here, we use the term functional expansion to refer to an increase in inferred metagenomic functional potential across the chronosequence, reflected by increased functional gene richness and shifts in the relative abundance structure of SEED-annotated functions. Specifically, we aimed to address three critical knowledge gaps: (i) Does the invisible below-ground ecosystem recover at the same rate as the visible above-ground flora? (ii) Do soil nutrient stoichiometries and microbial community structures converge over time, or do they continue to evolve over generational-scale timeframes? (iii) Which specific microbial taxa and metabolic functions act as indicators of successful restoration? By integrating vegetation surveys with detailed edaphic analysis, microbial community profiling, and metagenomic sequencing, our research provides a comprehensive view of ecosystem recovery at a landscape scale.

## Materials and methods

### Study site and land history

The Salisbury Plain site falls under the management of the UK Ministry of Defence and represents an area of *ca*. 38 000 ha. As a result of its use as a military training area for over a century, much of the landscape has remained inaccessible for agriculture beyond extensive livestock grazing and thus has not been subject to the agricultural intensification which destroyed calcareous grasslands across much of Western Europe over the twentieth century. Salisbury Plain thus contains Western Europe’s largest remaining fragment of lowland calcareous grassland and forms a unique landscape for exploring spatiotemporal patterns and processes in this habitat. Approximately 37% of the current military training area consists of high-quality, species-rich calcareous grassland communities, with the remainder being a mix of mesotrophic grassland, agriculturally improved pastures, and arable land. The land history classifications determined previously [11] from a time series of historic maps were used to determine sites suitable for analysis in this study. In total, seven arable, six recent, five middle, and seven ancient sites were chosen.

The minimum ages of unimproved grassland are as follows: (i) Arable, 000 years denotes areas lost to agricultural improvement and currently managed for arable crops; (ii) Recent, 023 years, grassland from 1985 to 1996; (iii) Middle, 067 years, grassland from 1930 to 1967; and (iv) Ancient, 143 years, grassland from 1840 to 1880. Because land history records do not extend beyond ~1840, the Ancient category represents a minimum age, and earlier site histories and potential heterogeneity cannot be fully resolved.

Soil samples were collected in January 2013. Per site, one sample was derived from the homogenization of five subsamples, collected along a 100-m linear transect using 5-cm virgin-plastic corers inserted into the soil to a depth of 15 cm (unless encountering an impenetrable chalk horizon). From each transect, soil from below the organic horizon was homogenized and stored in clean plastic bags and transfer to the laboratory for subsequent processing and storage at −20°C.

### Vegetation survey

Vegetation surveys were conducted in summer 2013 [11]. Briefly, for each discrete classification (Arable, Recent, Middle, and Ancient), the cover of vascular plant species was recorded from five square quadrats (each 2 m ×2 m) placed at 20-m intervals along the same 100-m transect used for soil sampling. Cover of all vascular plant species was recorded using the DAFOR scale [16]. DAFOR categories were converted to ordinal abundance scores (Dominant = 5 to Rare = 1; Present = 0.1) following common practice in ecological field studies, allowing calculation of relative abundance for Shannon diversity. Although semi-quantitative, this approach preserves species rank abundance relationships.

### Soil physicochemical properties

Subsamples of soil were sent to the commercial soil analysis provider NRM-Cawood Scientific, Bracknell, UK, and used to determine soil pH, total and organic carbon (% dry soil), phosphorus (mg/kg dry soil), potassium (mg/kg dry soil), magnesium (mg/kg dry soil), and soil texture percentages (clay <0.002 mm, silt 0.002–0.05 mm, sand 0.05–2.00 mm). Analysis of soil moisture (%), organic matter by loss on ignition (LOI % dry soil), and total and organic nitrogen (% dry soil) were undertaken at the UKCEH Centralised Chemistry laboratories (Lancaster) using established protocols [17]. C:N ratios were calculated from organic carbon and nitrogen values.

### Phospholipid fatty acid analysis

Phospholipids were extracted from 1.5 g soil fresh weight according to a previously published methods [18]. The following fungal and bacterial markers were summed for each sample and the fungi-to-bacteria ratio calculated. Fungal markers—C18:1ω9c, C18:2ω6c, and C18:2ω9t. Bacterial markers (Gram+ branched, Gram− monoenoics, cyclopropyl, actinomycetes)—‘i-C15:0’, ‘a-C15:0’, ‘i-C:16:0’, ‘i-C17:0’, ‘a-C17:0’, ‘i-C18:0’, ‘a-C18:0’, ‘C16:1ω7c’, ‘C16:1ω9c’, ‘C17:1ω10c’, ‘C18:1ω11c’, ‘C18:1ω11t’, ‘cy-C17:0*’, ‘9,10-cy-C19:0’, ‘11,12-cy-C19:0’, ‘10Me-C16:0’, ‘10Me-C17:0*’, and ‘10Me-C18:0*’.

### DNA extraction

Each sample was defrosted and DNA was extracted from 0.2 g of sample using the Mobio PowerSoil DNA extraction kit (Mobio, USA) following manufacturer’s instructions. Briefly, samples were lysed at 25 Hz for 20 min using a TissueLyser II (Qiagen, Germany). Purified DNA was eluted in 100 μl of elution buffer. DNA purity was assessed using a NanoDrop 8000 spectrophotometer (Thermo Fisher, UK), and concentration was measured using the Qubit dsDNA kit (Thermo Fisher). DNA was archived at −20°C at UKCEH, Wallingford, prior to amplicon and metagenomics analysis.

### Metagenomic sequencing and data processing

Library preparation and sequencing were conducted by Mr DNA (Texas, USA). Samples underwent 2 × 150 bp shotgun metagenomic sequencing on a HiSeq System (Illumina), targeting a depth of at least 4 Gb raw data per sample. The process generated a mean number of 9.8e+06 raw reads per sample.

Data processing followed analyses pipelines implemented using the Snakemake [19] workflow management system v7.8.2. Illumina adaptor sequences were trimmed and reads were filtered to a minimum quality score of 25 using Trim Galore v0.6.5. Reads mapping to the human reference genome (GRCh38) were removed. FastQC [20] and MultiQC [21] were used to assess the quality metrics of the preprocessed reads. To profile the community composition, SingleM v0.16.0 [22] was used to determine the percentage of archaea, bacteria, and non-prokaryotes which includes fungi and other eukaryotes. Taxonomic composition was profiled using SingleM within a reproducible workflow implemented in Snakemake. For each sample, paired-end reads were analysed with *singlem pipe* against the curated SingleM metapackage, which was downloaded automatically using *singlem data* to ensure consistent reference versions across runs. Outputs from all samples were subsequently integrated using *singlem summarise* to produce combined abundance matrices and species-by-site coverage estimates.

### Assembly and functional and taxonomic annotation

Reads were assembled into contigs using Megahit v1.2.9 [23], which formed the basis for downstream annotation and analysis (Supplementary Table 1). For functional analysis, open reading frames were predicted using Prodigal v2.6.3 [24] and annotated using EggNOG-mapper v2.1.9 [25] against the v6.0 database and the SEED classification database [26]. Gene abundance was calculated using featureCounts [27]. In this study, functional composition refers to the relative abundance structure of these SEED-annotated categories within each sample, based on gene counts normalized for downstream comparative analyses. Herein, all references to microbial function relate to inferred metagenomic functional potential, derived from gene content, rather than direct measurements of gene expression, enzyme activity, or process-level rates. Accordingly, functional expansion in this study refers to an increase in inferred metagenomic functional potential across restoration age, evaluated primarily through functional gene richness and gene diversity. To provide a high-resolution taxonomic profile independent of genome size biases, we processed the metagenomic shotgun sequencing data using SingleM to identify and extract fragments of 14 universal single-copy marker genes (e.g. rplB, rpsC) from the raw reads. These sequences were clustered into operational taxonomic units (OTUs) and taxonomically assigned using the SingleM default database. Taxonomic levels were parsed from the resulting strings and best-name identifiers were generated by selecting the most specific classified level (from species to phylum) for each OTU to ensure informative labelling of uncultured or novel taxa. The community data were normalized by mean coverage implemented within singleM (estimated genome equivalents) and integrated with the soil chronosequence metadata. The fraction of metagenomic reads derived from prokaryotic cells (Bacteria and Archaea) was estimated using *singleM prokaryotic_fraction*.

### Invertebrate isolation

Samples for extraction of soil invertebrates were collected in October 2015 (2 years after soil sample collection and vegetation surveys). Consistent with a previously published method [28], four soil plugs were cut per site for Tullgren extraction, a design considered appropriate for site-level comparative analysis, and more intensive sampling would be required for scarce or larger-bodied taxa. Briefly, four 10-cm-diameter cores were taken at each site by placing a plastic ring on the surface and using a knife to cut the surface vegetation and subsurface roots. The turf and first 10 cm of soil were then lifted out as a plug and placed in clean plastic bags. Within 24 h, these plugs were positioned in Tullgren funnels and all invertebrates leaving the sample were captured and stored in 100% ethanol. The invertebrate catch were subsequently removed from the storage ethanol, dried, and placed into Mobio PowerSoil DNA extraction kit (Mobio) for DNA extraction following manufacturer’s instructions.

### Amplicon sequencing

Amplicon libraries were constructed according using a dual indexing strategy [29] with each primer consisting of the appropriate Illumina adapter, 8-nt index sequence, a 10-nt pad sequence, a 2-nt linker, and the amplicon specific primer. For the V3–V4 region: 16S rRNA gene (bacteria) CCTACGGGAGGCAGCAG and GGACTACHVGGGTWTCTAAT [29], ITS2 (fungi) GTGARTCATCGAATCTTTG and TCCTCCGCTTATTGATATGC [30], and COI (invertebrates, predominantly Arthropoda, Mollusca, and Nematoda) GGWACWGGWTGAACWGTWTAYCCYCC and TAIACYTCIGGRTGICCRAARAAYCA [31]. The pooled libraries were sequenced separately on a MiSeq System (Illumina) with V3–600-cycle flow cells and demultiplexed at UKCEH. Sequence tables for 16S and COI amplicons were generated according to a previously published method [32], and the ITS fungal amplicon sequence table was produced using PIPITS [33]. After initial quality checks using R package *microeco* [34], sample reads were rarefied to the minimum sample read number per amplicon of 2385, 4792, and 8184 for COI, 16S, and ITS reads, respectively.

### Statistical analysis

Statistical analyses assessed the recovery of soil biotic properties across the restoration chronosequence. R package *vegan* [35] produced alpha-diversity metrics, including Shannon index, Species richness, and Pielou’s evenness, to quantify structure. The co-variance of measured edaphic variables were assessed with linear models. Variance inflation factors (Supplementary Table 2) indicated moderate to high multicollinearity among edaphic predictors, ranging from 2.7 to 48.5. The highest values were associated with soil organic matter (loss on ignition, VIF = 48.5) and organic nitrogen (VIF = 43.4), reflecting their strong correlation. Elevated VIF values were also observed for clay (13.6), phosphorus (10.6), and magnesium (10.0); pH (5.2), moisture (5.2), and potassium (4.2) showed moderate collinearity. In contrast, the C:N ratio exhibited relatively low multicollinearity (VIF = 2.7). The following variables were retained due to their distinct ecological relevance: C:N, organic nitrogen, magnesium, phosphorus, potassium, pH, moisture, and clay. Functional analyses were based on SEED-annotated metagenomic gene profiles and therefore reflect inferred functional potential rather than realized microbial activity. Within this framework, functional gene richness and functional composition were treated as the primary descriptors of functional expansion and correlations with LOI were used as an independent analysis linking functional patterns to soil development. Simultaneously, functional compositional differences between restoration ages were visualized using principal component analysis (PCA) with vectors of edaphic drivers added. Functional annotations across hierarchy levels (SEED levels 1–4) were reshaped into long format and summarized by restoration age. For each function and time point, mean relative abundance was calculated across samples. To identify functional genes exhibiting consistent directional change over restoration, mean relative abundances were compared across the four time points (000, 023, 067, and 143 years). Functional genes were classified as emergent if they were absent at baseline (mean abundance = 0 at 000 years) and exhibited monotonic increases thereafter, and as declining if they were present at baseline and exhibited monotonic decreases across successive time points, with a net decrease from 000 to 143 years. For each gene function, the magnitude of change was quantified as the difference in mean relative abundance between 143 and 000 years. For visualization, genetic functions were grouped by SEED hierarchy level and direction of change (emergent or declining), and the top functions within each group were selected based on the absolute magnitude of change. Mean relative abundances at each time point were displayed as a heatmap, with restoration age on the *x*-axis and functions on the *y*-axis, ordered by magnitude of change within each ontology level. Tile colour represents mean relative abundance, after log₁₀-transformation, to enhance contrast among low-abundance functions. This approach emphasizes both the temporal dynamics and ontological structure of functional shifts during ecosystem restoration.

We defined two distinct successional trajectories to characterize microbial colonization. Pioneer or early emergent taxa were those with zero detected coverage at the initial arable stage (000y) that reached a mean coverage threshold of >0.05 by the early restoration stage (023y). Also, monotonically increasing taxa demonstrated a consistent, non-decreasing trend in mean coverage across all four time points to 143y, representing stable, long-term colonizers of the developing soil ecosystem.

Direct correlations between genetic functions and soil organic matter (LOI), a key soil health metric, were assessed using Spearman’s rank correlation coefficient. To identify microbial functions that tightly track soil development, we performed a correlation-based analysis between SEED functional gene abundances and soil organic matter (LOI %). Given the asymptotic accumulation of LOI observed across the chronosequence stages, Spearman’s rank-order correlation (rho) was utilized to capture monotonic relationships while accounting for sample-level variance (*n* = 25). Prior to correlation analysis, functional groups were pre-filtered to include only those exhibiting a consistent emergent or declining trajectory across the successional stages. Multiple testing was accounted for using the Benjamini–Hochberg false discovery rate adjustment, with significance set at *P* <0.05. To visualize the transition of functional drivers across different scales of metabolic complexity, the top eight strongest correlates were selected for each hierarchical SEED level (levels 1 through 4). Results were visualized using a lollipop plot, where the distance from the zero-intercept and colour intensity denote the strength and direction of the successional coupling.

## Results

### Responses across the chronosequence

**Figure 1:**
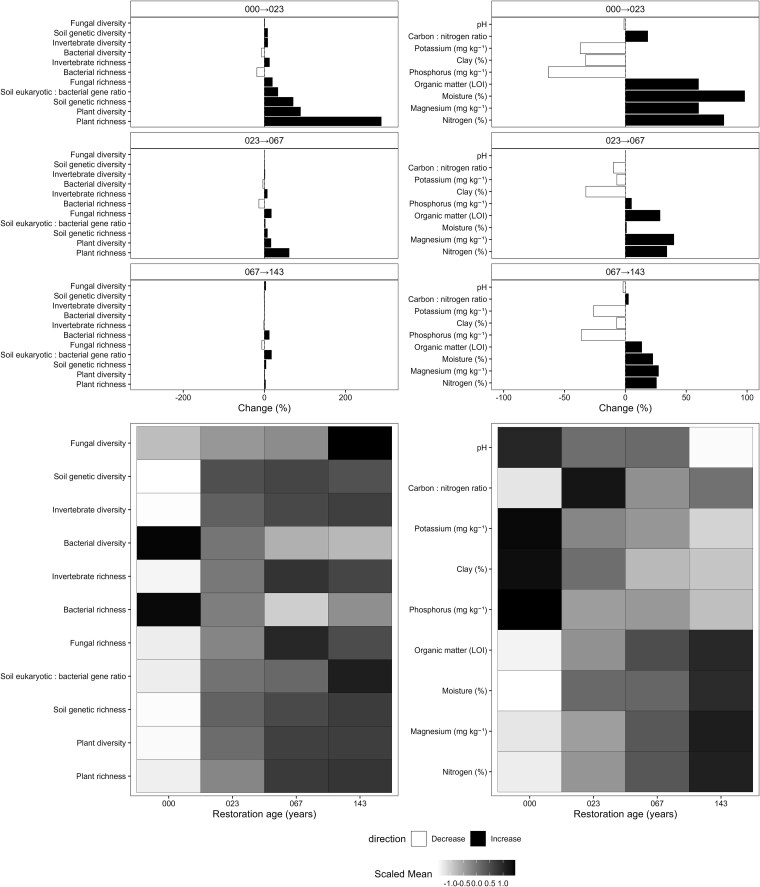
System response dynamics across a restoration chronosequence. Response speed of biological (left) and edaphic (right) variables shown as percent change between successive restoration intervals (000–023, 023–067, and 067–143 years) and standardized trajectories of the same variables across the full chronosequence. Bars indicate the magnitude and direction of change within each interval, while heatmaps illustrate relative temporal trajectories across restoration ages. Variables are ordered by the absolute magnitude of percent change across the chronosequence to facilitate comparison of response strength and temporal dynamics.

To understand how the passive restoration of these soils might influence various soil and vegetative properties, we assessed several factors over the course of the recovery period. Analysis of the rates of change (Fig. 1) showed that the magnitude of change varied across the chronosequence, with the largest shifts occurring early in restoration. The highest rates of change occurred in the initial, 000-to-023-year, interval, driven by very large increases in vegetation richness and decreases in soil phosphorus. However, the system did not stabilize after this initial burst. Between 067 and 143 years, significant increases continued to occur (Supplementary Figs. 1 and 2, and  Table  3) particularly in the variables of soil organic matter (LOI; 20 to 23%), organic nitrogen (0.8% to 1.0%), magnesium (112 to 143 mg/kg), and the eukaryotic:bacterial gene ratio (0.48 to 0.57). This indicates that even after nearly seven decades of recovery, the soil ecosystem remained distinct from 143-year ancient grassland stage.

### Vegetative and edaphic recovery is decoupled

The restoration trajectories revealed a decoupling between plant community reassembly and soil chemical recovery (Fig. 1). Soil chemical variables exhibited a slower, continuous recovery, where soil organic matter (LOI) and nitrogen (N) concentration increased monotonically from the arable baseline to the 143-year ancient grasslands (Fig. 1). This increase in organic matter and nitrogen likely reflects the progressive accumulation of increasingly complex organic matter pools during succession, in which nitrogen is contained within stabilized or recalcitrant forms. In contrast, legacies of agricultural fertilization persisted with insignificantly elevated levels of phosphorus (P) and potassium (K) in 023- and 067-year restoration sites compared to ancient grasslands (Supplementary Fig. 2 and Table  3).

Vegetation richness and diversity (Shannon index) responded more rapidly than soil chemical properties after agricultural cessation (Fig. 1), increasing significantly to 023 years (~300%); a further increase in richness occurs 023 to 067 years and then gradual, but nonsignificant, increases occur between 067-to-143-year stage (Supplementary Fig. 1).

### Microbial variance over time

The recovery of the soil microbiome followed a trajectory distinct from that of the vegetative community (Fig. 1), characterized by an inverse relationship between taxonomic diversity and genetic functional potential. Contrary to the trajectory observed in plant communities, soil bacterial diversity decreased with restoration age. Both bacterial richness and diversity were significantly higher in arable soils (000 years) (Supplementary Fig. 1). The cessation of agricultural practices resulted in a sharp decline in bacterial richness during the initial restoration phase, with richness remaining lower and relatively stable across the older grasslands. Despite the reduction in taxonomic bacterial richness, the inferred metagenomic functional potential of the soil microbiome expanded with ecosystem age. Functional gene richness was lowest in the arable sites but increased significantly within the first 23 years of restoration (Supplementary Fig. 1). This pattern represents the primary sense in which we refer to functional expansion across the chronosequence. Unlike bacterial richness, which dropped and plateaued, functional genetic richness remained elevated in the older grassland soils, suggesting that the communities become genetically more complex over time despite being composed of fewer bacterial taxa. Concurrently, the composition of the soil community exhibited a monotonic shift towards non-prokaryotic dominance across the chronosequence. The SingleM-derived eukaryotic to bacterial gene ratio (Fig. 1), utilized as a metric for broad structural change, recorded lowest ratios in the arable soils and significant increases with time (*singlem_fb_ratio*, Supplementary Fig. 1). This indicates a continuous proliferation of fungal and other non-prokaryotic organisms, with the highest ratios observed in the ancient (143-year) samples.

### Microbial community structure and emergent taxa

**Figure 2:**
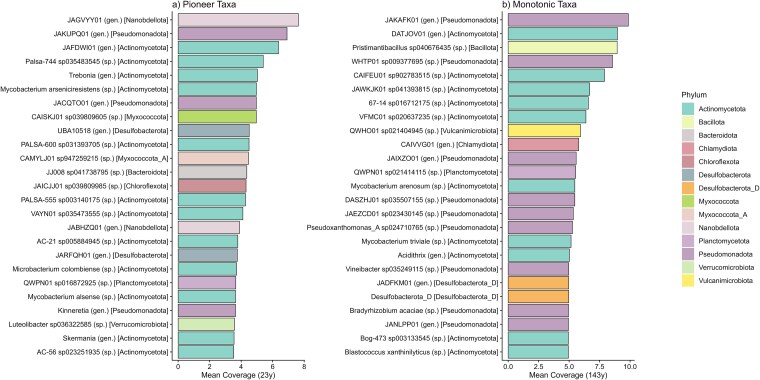
Successional dynamics of microbial taxa across the soil chronosequence as determined by SingleM marker gene analysis. Taxa are ranked by mean coverage (estimated genome equivalents) and labelled with the most specific classified taxonomic level, with the associated phylum provided in brackets. (a) Pioneer taxa: the top 25 taxa that were undetected at the 0-year (arable) stage but became established by year 23 of restoration. (b) Monotonically increasing taxa: the top 25 taxa that exhibited a continuous increase in abundance from the arable stage through to the 143-year established grassland stage. Bars illustrate the broad phylogenetic shifts accompanying the transition from agricultural land to established natural grassland. Taxa labels include taxonomic rank indicators: (sp.) species, (gen.) genus.

Despite the reduction in taxonomic richness, the inferred genetic functional potential of the microbiome expanded over time. Soil genetic richness showed a significant positive response during the 000-to-023-year interval (Fig. 1, Supplementary Fig. 1), this shift was congruent with the early establishment of emergent taxa that were absent in arable soils (Fig. 2a). By year 023, the microbial community exhibited specific pioneer genera and species with the top five taxa belonging to *Pseudomonadota* and *Actinomycetota*, including a genus of the episymbiont *Nanobdellota*. Over the course of the chronosequence, taxa that demonstrate monotonic increase (Fig. 2b) include specific genera of *Pseudomonadota*, *Actinomycetota*, and *Pristimantibacillus* sp. The prominence of *Actinomycetota* and *Bacillota* suggests a functional reorientation towards the decomposition of complex organic matter and resource-conservative life strategies.

### Distinct functional states and metabolic shifts

**Figure 3:**
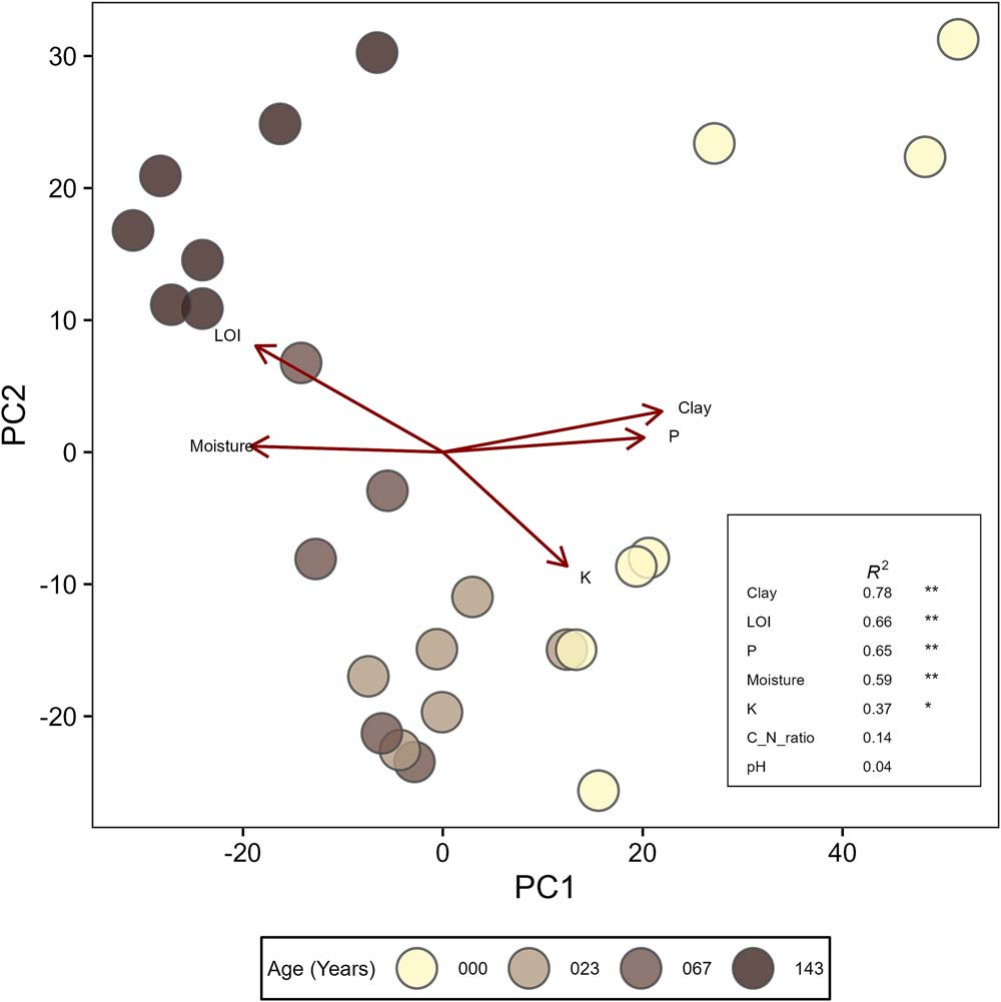
PCA of microbial functional groups with significant environmental drivers. Points represent functional composition, coloured by chronosequence age. Vectors represent significant environmental variables (*P* < .05) based on envfit analysis. Inset table shows goodness-of-fit (*R*^2^) and significance codes for all edaphic parameters.

To understand how the recovery of the soils influenced microbial community functions, we used an ordination method which indicated that soil functional composition did not converge on a single recovered state but instead differed across chronosequence stages. This revealed clear clustering based on restoration age, with the first two principal components explaining 18.6% of the total variance (Fig. 3). The first two principal components indicated separation between the arable and early restoration (000- and 023-year) sites from the ancient grasslands (143 years). This functional separation was associated with opposing environmental vectors, where arable and early restoration sites were associated with legacies of agricultural improvement (K, P, and clay content), while ancient sites were congruent with vectors associated with ecosystem stability and organic matter sequestration (LOI, moisture, and C:N ratio). pH, a known microbial master variable, did not emerge as a significant driver of functional composition (in-laid table, Fig. 3 and Supplementary Fig. 2), highlighting soil organic matter accrual as a prominent edaphic correlate of functional composition in this system. The 67-year sites occupied an intermediate ordination space, further indicating that functional composition maturation is not achieved after six decades of recovery.

### Emergence and loss of specialized functions and pathways

**Figure 4:**
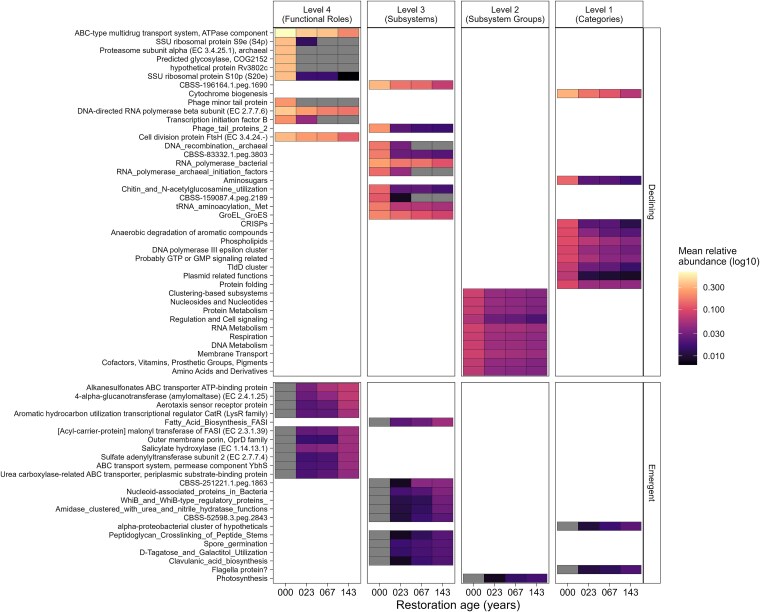
Ontology-resolved temporal dynamics of microbial functional potential across restoration. Heatmap showing mean relative abundances of SEED-annotated microbial functions across restoration age (000, 023, 067, and 143 years). Functions are grouped by SEED hierarchy level (columns) and by direction of change over time (rows), classified as emergent (net increase from 000 to 143 years) or declining (net decrease). Within each hierarchy level and direction, functions are ranked by the magnitude of absolute change in mean relative abundance between 000 and 143 years, and the top functions are displayed. Tile colour indicates mean relative abundance at each time point, shown on a log₁₀-transformed colour scale to enhance contrast among low-abundance functions. This representation highlights both the timing and ontology of functional shifts during ecosystem restoration.

We identified a suite of functional genes that exhibited monotonic increases or decreases in abundance with restoration age (Fig. 4). Restoration of soil led to notable decreases in the abundance of genes associated with membrane transport (ABC-type multidrug transport system), ribosomes (SSU ribosomal proteins), archaeal protein recycling (archaeal protease subunits), DNA repair (glycosylase), and phage proteins (phage tail proteins). Overall, the recovery of soil condition leads to a reduction in genes associated with growth, maintenance, and disease, along with broader reductions in the subsystems associated with metabolism of proteins, RNA, and DNA. While these functions decreased with restoration age, monotonic increases in genes and pathways along the chronosequence were also observed. Subsystems for photosynthesis and motility were more abundant in older soils, consistent with the establishment of mobile, autotrophic, or mixotrophic communities. Increases in flagella and aerotaxis sensor receptor proteins enable bacteria to navigate towards optimal oxygen concentrations, maximizing energy production. Flagella-associated functions have been previously correlated with increasing organic matter [36]. Fatty acid biosynthesis genes, essential for cell membrane production and membrane fluidity, may support adaptation to environmental stress. These functional gene increases reveal that as soil structural complexity increases with restoration age, soil genetic functions increase. Additionally, the ability to acquire organosulfur compounds becomes more important with restoration age (alkane sulfonate ABC transporters), suggesting that sulphur availability may be a rate-limiting factor. Increases in 4-α-glucanotransferase suggest greater utilization of complex carbohydrate metabolism, indicating a shift to the utilization of complex substrates as soil organic matter content increases.

### Microbial functional correlates of soil organic matter accumulation

**Figure 5:**
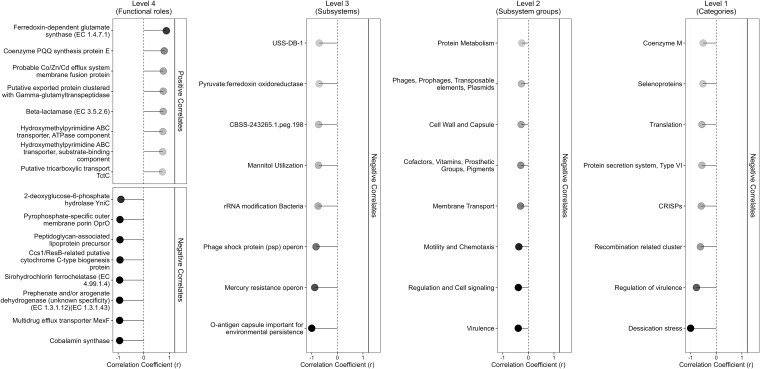
Key functional microbial correlates of soil organic matter (LOI) accumulation. Correlates were identified by Spearman’s rank correlation (rho) between the relative abundance of SEED functional groups and soil organic matter content (loss on ignition, LOI %) across all soil samples (*n* = 25). The lollipop plot displays the top eight significantly correlated functions (*P* < .05) per hierarchical SEED level (levels 1–4). The position and opacity of the points represent the correlation coefficient with LOI.

Soil organic matter (SOM), measured here as LOI, is broadly and consistently identified as a central soil health parameter [37, 38]. pH is established as a master variable influencing microbial community composition and function [39], and within this dataset no significant change in pH with restoration age was observed (Fig. 3 and Supplementary Fig. 2), providing an opportunity to examine functional gene categories that covary with soil organic matter as an indicator of soil development and condition. Spearman’s rank correlation analysis (Fig. 5) identified inverse correlations between SOM and genes, subsystems, and categories associated with metabolism (proteins, RNA, and DNA), growth (respiration, ribosomes, cell division, and cell walls), and disease and genetic transfer (CRISPRs and phages). In contrast, strong positive SOM correlations were found with genes for stress reduction (PQQ synthesis, Co/Zn/Cd efflux), antibiotic resistance (beta-lactamase), and auxotrophy (HMP ABC transporters).

## Discussion

The results of this calcareous grassland study challenge the assumption that ecosystem recovery is a synchronous process where below-ground health mirrors above-ground biodiversity. Instead, we observe a complex, multi-phasic recovery where soil properties lag significantly behind the recovery of vegetation.

### Decoupling of vegetation and soil diversity recovery

The results of our study reveal a disconnect between the recovery of above-ground floristic diversity and below-ground edaphic genetic function. Because our study is based on a space-for-time chronosequence rather than repeated sampling of the same sites through time, the observed recovery patterns should be interpreted as consistent with, rather than definitive evidence of, particular ecological mechanisms. Congruent with earlier work [11, 40–42], we found that increases in plant diversity and species richness are rapidly achievable. In our study, vegetation richness increased significantly in the first 23 years and continued to rise before stabilizing from 067 years, suggesting a relatively rapid recovery of taxonomic diversity within a human lifetime, compared with the slower recovery of soil properties. However, broad diversity metrics can obscure underlying functional deficits. As observed in 2014 [11] within this same landscape, even in well-connected landscapes, ancient grasslands show distinct vegetation communities and traits, suggesting that natural regeneration to a full ancient calcareous grassland community takes over a century. While the richness of the vegetation may approach ancient levels relatively quickly, the functional assembly of the plant community remains distinct for much longer. This delay mirrors the persistent legacy effects and slow convergence we observe in the soil ecosystem, which exhibited continued diversification and functional evolution well beyond 067 years. Thus, we observe a two-speed recovery model with a rapid biological reassembly of plant taxonomic richness, followed by a protracted, multi-generational recovery of plant functional traits, soil chemistry, and microbiome architecture. Another potential explanation for the observed disparity may be due to the increased organic matter carrying capacity of calcareous soils within this landscape. For example, previously studies reported >20% OM in similar soils [43]. We will, however, need future empirical assessments to disentangle these complex interactions.

### The persistence of agricultural legacies and carbon dynamics

The contrast in recovery rates is associated with the persistence of agricultural legacies [8, 44]. The elevated levels of available P and K in the younger restoration sites indicate that the eutrophication caused by historical fertilization takes decades to attenuate. This nutrient enrichment may help explain the delayed establishment of the stress-tolerant, oligotrophic microbial specialists characteristic of ancient soils [45] and acts as a potential mechanism explaining the observation that in many studies, sites appear to stall at a stable, mesotrophic community for long periods [8, 11, 46]. Conversely, the accumulation of C and N follow a monotonic trajectory that did not saturate even after 067 years. Increasing total organic carbon and nitrogen should not be interpreted as evidence of increasingly copiotrophic conditions with more readily available nutrients for microbial growth. As succession proceeds, carbon and nitrogen are increasingly incorporated into complex organic matter pools [47], such that total carbon and nitrogen become decoupled from nutrient bioavailability. Older sites, despite higher organic carbon and nitrogen content, are associated with taxa indicative of slower-growing, resource-conservative life strategies [48]. These patterns suggest that the carbon sequestration potential of calcareous grasslands is not a rapid solution but a long-term service that shows no evidence of saturation within the first 143 years of recovery and may represent a multi-generational process. Consequently, carbon credit schemes or conservation policies based on short-term restoration (<30 years) may severely underestimate the time required to restore the full soil carbon sink in these systems.

### Functional redundancy at high microbial diversity

A key finding of our study is the inverse relationship between bacterial richness and ecosystem maturity. Here, as per previous studies, arable soils supported the highest bacterial richness and diversity [49, 50], likely representing a bloom of fast-growing, disturbance-adapted copiotrophs fuelled by fertilizer inputs and labile carbon [51]. In contrast, natural regeneration appears to impose strong environmental filtering, reducing bacterial taxonomic richness and diversity while simultaneously increasing inferred metagenomic functional potential, particularly functional gene richness [52]. We postulate that this transition is consistent with a shift towards more functionally differentiated and specialized communities. This specialization is further implied by the monotonic increase in the non-bacterial-to-bacterial ratio and the increase of Actinobacteria and Bacilli along the chronosequence. The rise of Actinobacteria and fungi is consistent with a functional reorientation towards the decomposition of more recalcitrant organic matter, in line with their known ecological roles.

### Functional maturation and environmental feedback loops

Our findings reveal a clear coupling between vegetation recovery, soil moisture, and the accumulation of organic matter, which mirrors shifts in inferred metagenomic functional expansion. We interpret these patterns through the lens of the plant–soil feedback framework [53]. Under this model, the re-establishment of specific plant traits actively conditions the edaphic environment, driving successional trajectories. Specifically, our analysis suggests that as root exudates, litter, and organic matter accumulate, they exert selective pressure that increasingly favours specialized metabolic pathways over generalized ones. Specialization along the chronosequence also includes the enrichment of genes associated with motility and survival, reflecting the community’s adaptation to the unbuffered physical stresses and spatial heterogeneity of natural environments. This contrasts with the homogeneity of managed arable soils, where frequent tillage and inputs cushion microorganisms against physicochemical variation. This trajectory mirrors trade-offs previously described [54, 55], where a shift from r-strategist (copiotrophic) dominance in disturbed, nutrient-rich soils to K-strategist (oligotrophic/stress-tolerant) dominance in stable, late-successional ecosystems was observed. Soil pH, widely regarded as the master variable determining global soil microbial community structure [49, 50], did not change in this system, usefully decoupling it as a driver of observed genetic functional change.

### Incomplete functional convergence and conservation implications

This study demonstrates that below-ground recovery remains incomplete within the timescale captured by the chronosequence. The ordination and rate-of-change analyses demonstrated that 67-year-old grasslands are genetically functionally distinct from 143-year-old ancient sites. The trajectory of edaphic change, particularly in C, moisture, and Mg, suggests that restoration is a continuous process spanning multiple human generations [56]. This challenges binary definitions of restored versus degraded land [57]. Therefore, conservation policy must recognize that within a temperate calcareous landscape, 60-year-old regeneration sites are valuable but transitional ecosystems, requiring continued protection to allow the slow accumulation of the functional complexity and specialized metabolic depth which characterizes these ancient soils.

### Conclusion and future research

Looking forward, this 143-year chronosequence necessitates a paradigm shift in how we value and manage restoring calcareous grassland landscapes, moving beyond the assumption that ecosystem recovery is a rapid or synchronous process. The persistence of agricultural nutrient legacies and the lack of functional convergence even after seven decades demonstrate that current conservation frameworks, often bound by short-term funding cycles, are ill-equipped to capture the full trajectory of edaphic recovery. To address the temporal gap between rapid vegetation recovery and slower soil functional expansion, future restoration studies could examine approaches that extend beyond passive regeneration. Our results are consistent with the possibility that dispersal limitation and persistent nutrient legacies could constrain the reassembly of specialized microbial communities. A future hypothesis to test is targeted soil inoculation whereby early restoration sites receive small amounts of donor soil from long-established grasslands to reintroduce oligotrophic taxa. Experimental studies have demonstrated that whole-soil inoculation can help overcome dispersal barriers and steer plant and soil community assembly towards reference states, accelerating the development of late-successional functions [58]. Although not tested here, such approaches may enhance the recovery of tightly coupled nutrient cycling processes that often remain incomplete after decades of spontaneous regeneration [59].

The persistence of elevated phosphorus in younger sites suggests that nutrient legacies may act as a bottleneck for microbial successional speed. Future research should investigate whether mitigating these legacies, through targeted nutrient reduction or sequestration strategies, can accelerate microbial reassembly trajectories.

Within our study protists were not explicitly profiled, given their important roles as microbial predators and regulators of nutrient turnover, incorporating protist community dynamics would be a valuable next step in understanding whole-soil food web recovery during restoration.

Our findings highlight a potential mismatch between rapid vegetation recovery and slower soil functional maturation. Specifically, our findings suggest that future monitoring frameworks could consider incorporating below-ground indicators alongside above-ground metrics. For example, metagenomic assessment of genes associated with stress tolerance and carbon cycling could provide complementary indicators of functional development. More broadly, the continued increase in soil organic matter across this calcareous chronosequence suggests that carbon accrual operates over multi-decadal to centennial timescales. Collectively, this reinforces the importance of aligning restoration expectations with the protracted temporal dynamics of soil ecosystem recovery.

## Supplementary Material

Supplementary_material_wrag131

## Data Availability

Sequence data are available at NCBI-SRA under BioProject ID: PRJNA1424699. Scripts and data tables are available at https://doi.org/10.5281/zenodo.18632861.
