# Supplementary material for: Taxonomic filtering accompanies functional expansion during long-term soil restoration
Source: ISME J. 2026 May 22;20(1):wrag131. doi: 10.1093/ismejo/wrag131 (PMC13280953; doi:10.1093/ismejo/wrag131)
Supplement: Supplementary_material_wrag131 [file supplementary_material_wrag131.zip › Supplementary legends.docx]

**Supplementary figure 1. Successional dynamics of biological variables across a 143-year soil chronosequence.** Box-and-whisker plots represent the distribution of vegetation (richness, Shannon diversity, and Pielou’s evenness), invertebrate communities, microbial indices (including bacterial and fungal metrics, PLFA derived fungal-bacterial ratio and SingleM derived eukaryote-bacterial gene ratio), and genetic functions. Data points are jittered to show individual sample distribution. The colour gradient represents soil age, transitioning from light yellow (000y) to dark brown (143y). Different lowercase letters above boxes indicate significant differences between age groups based on Tukey’s Honestly Significant Difference (HSD) test (*P* < 0.05).

**Supplementary figure 2. Evolution of edaphic properties and soil texture over 143 years of soil development.** Box-and-whisker plots represent the distribution of soil nutrients (LOI%, organic Nitrogen%, C:N ratio, Phosphorus, Potassium, and Magnesium mg / kg), physical properties (Moisture% and pH), and soil texture (Sand, Silt, and Clay %). The colour ramp follows the chronosequence from light yellow (000y) to dark brown (143y). Data points are jittered to show individual sample distribution. The colour gradient represents soil age, transitioning from light yellow (000y) to dark brown (143y). Different lowercase letters above boxes indicate significant differences between age groups based on Tukey’s Honestly Significant Difference (HSD) test (*P* < 0.05).

**Supplementary table 1. Metagenome read assembly and mapping statistics.**

**Supplementary table 2. Variance inflation factors of measured variables.**

**Supplementary table 3. Mean values of measured variables across the chronosequence.**
