# Supplementary figures and images for: Taxonomic filtering accompanies functional expansion during long-term soil restoration
Source: ISME J. 2026 May 22;20(1):wrag131. doi: 10.1093/ismejo/wrag131 (PMC13280953; doi:10.1093/ismejo/wrag131)

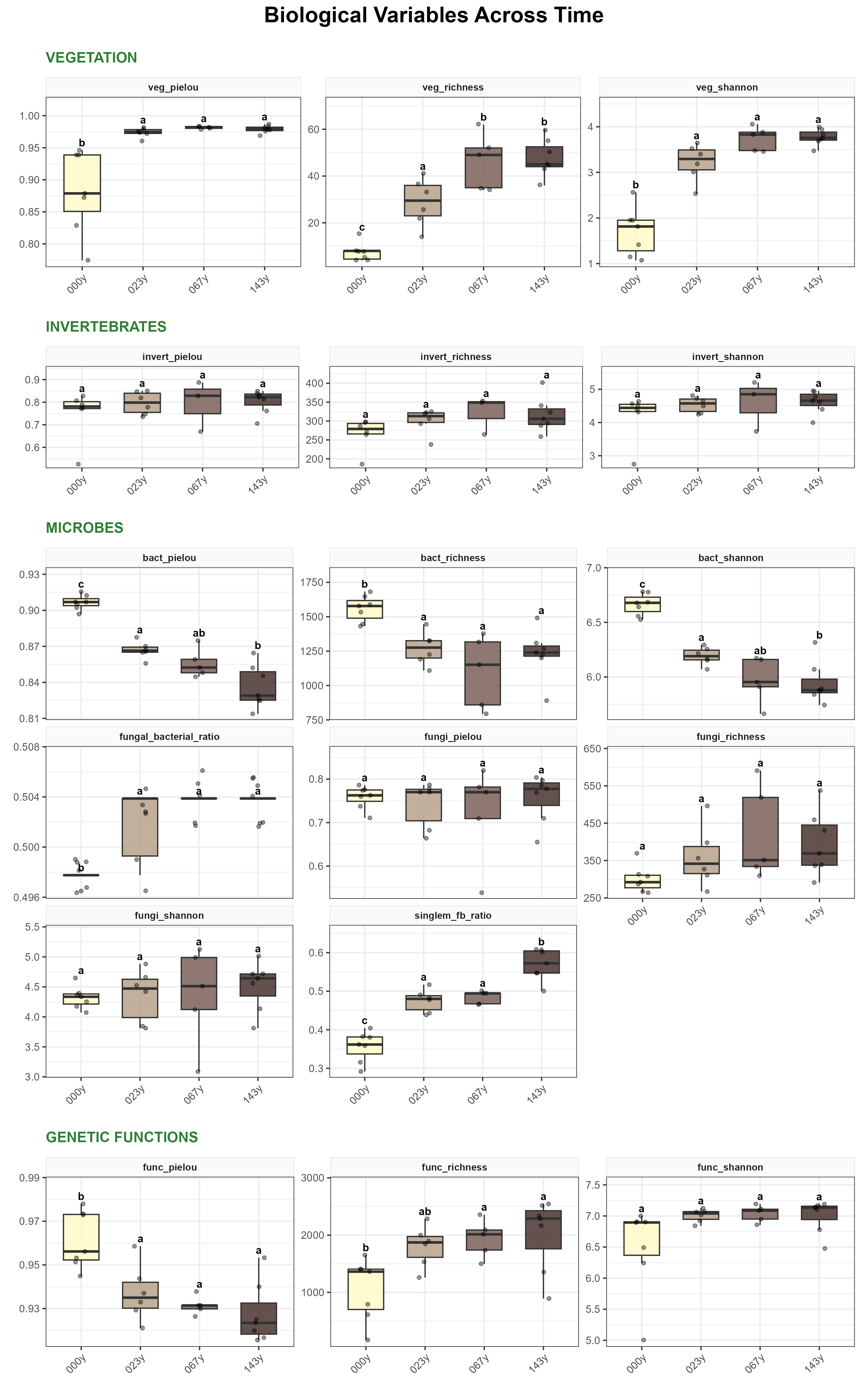

Supplement: Supplementary_material_wrag131 [file supplementary_material_wrag131.zip › Supp_Fig_1_Soil_Biological_Variables.tiff]

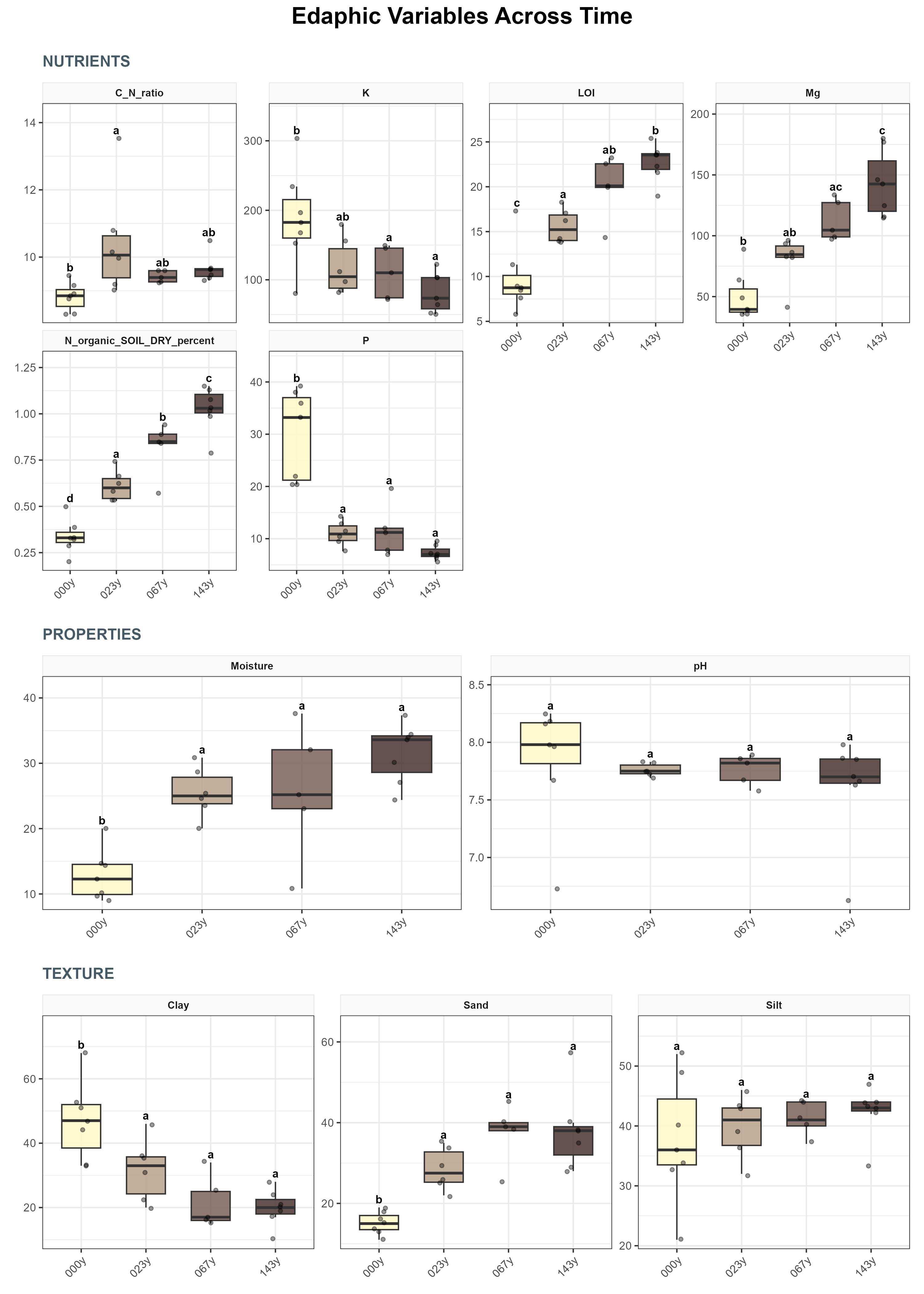

Supplement: Supplementary_material_wrag131 [file supplementary_material_wrag131.zip › Supp_Fig_2_Soil_Edaphic_Variables.tiff]
